# 3.N. Workshop: IMAgiNE EURO: Quality of maternity care in the WHO European Region during the COVID-19 pandemic

**DOI:** 10.1093/eurpub/ckac129.188

**Published:** 2022-10-25

**Authors:** Organised by: IMAgiNE EURO Study Group, Chair persons: Céline Miani (Germany), Marzia Lazzerini (Italy)

## Abstract

From 2020 the COVID-19 pandemic has deeply affected maternal and neonatal care. The implementation of governmental policy responses to limit the spread of the disease and the development of new hospital protocols have forced healthcare workers and women alike to adapt. The perception of the quality of hospital care by both groups has been at the core of the IMAgiNE EURO project, led by the WHO Collaborating Center for Maternal and Child Health (Trieste, IT). With a network of 18 countries and more than 40 institutions, this collaborative project has gathered so far data from more than 50 000 births and 5000 health workers, through two validated online surveys. It allows monitoring the quality of maternal and newborn care in four domains: the three domains of the WHO Standards for improving quality of maternal and newborn care in health facilities (namely provision of care, experience of care, and availability of human and essential physical resources) and the additional domain of key organisational changes related to the COVID-19 pandemic. Following multi-country analyses, and analyses at the regional and national level, the project findings show major gaps in the perceived quality of maternal and neonatal care in hospitals during the pandemic, as well as large variations in practices across different countries of the WHO European Region. In this workshop, Dr. Emanuelle Valente (WHO Collaborating Center for Maternal and Child Health, Trieste, IT) first introduces the methodological development of the project and the two validated data collection instruments. Dr. Marzia Lazzerini (WHO Collaborating Center for Maternal and Child Health, Trieste, IT) then presents the main results of the women's questionnaire for all countries. The focus will be on the Quality of Maternal and Newborn Care Index (QMNC Index) and how the different countries perform with regard to the four above-mentioned dimensions. Then, we carry on with two presentations focusing on specific topics relevant to maternity and newborn care. Dr. Ilana Chertock (College of Health Sciences and Professions, Ohio University, Athens, Ohio, USA) and Dr. Rada Artzi-Medvedik (Ben-Gurion University, Beersheva, Israel) will present findings on the factors influencing exclusive breastfeeding in healthcare facilities during the pandemic. Dr. Céline Miani (Institute of Public Health, Bielefeld University, Bielefeld, Germany) will discuss the topic of medicalisation, looking at the potential associations between individual and country-level factors and medicalisation of birth. We will conclude the workshop with a panel discussion on the implications of our research for policies and practice, and examples of the first steps already taken to bridge the gap between monitoring and implementation.

**Key messages:**

• This workshop will present two new, validated tools to monitor the quality of maternal and newborn care in WHO European Region, from the perspective of the women and of the healthcare providers.

• It will also provide an overview of key findings so far, highlighting inequalities and gaps in the quality of maternal and neonatal care at hospital level in the WHO European Region.

